# Preliminary Evidence Suggests That a 12-Week Treatment with Tirzepatide Plus Low-Energy Ketogenic Therapy Is More Effective than Its Combination with a Low-Calorie Diet in Preserving Fat-Free Mass, Muscle Strength, and Resting Metabolic Rate in Patients with Obesity

**DOI:** 10.3390/nu17071216

**Published:** 2025-03-30

**Authors:** Luigi Schiavo, Biagio Santella, Monica Mingo, Gianluca Rossetti, Marcello Orio, Luigi Cobellis, Attilio Maurano, Antonio Iannelli, Vincenzo Pilone

**Affiliations:** 1 Department of Medicine, Surgery and Dentistry “Scuola Medica Salernitana”, University of Salerno, 84081 Baronissi, Italy; bsantella@unisa.it (B.S.); mmingo@unisa.it (M.M.); 2 NBFC, National Biodiversity Future Center, 90133 Palermo, Italy; 3 General and Bariatric Surgery Unit, Abano Terme Policlinic, 35031 Padova, Italy; gianlucarossetti@yahoo.it; 4 Medical and Diabetological Center CMSO, 84123 Salerno, Italy; marcello.orio@gmail.com; 5 Unit of General Surgery, Casa Di Cura “Prof. Dott. Luigi Cobellis”, 84078 Vallo della Lucania, Italy; luicobellis@yahoo.it; 6 Digestive Endoscopic Unit, Ruggiero Clinic, 84013 Cava de Tirreni, Italy; amauran@icloud.com; 7 Digestive Surgery and Liver Transplantation Unit, Archet 2 Hospital, Centre Hospitalier Universitaire de Nice, 06001 Nice, France; iannelli.a@chu-nice.fr; 8 Université Côte d’Azur, 06001 Nice, France; 9 Team 8 “Hepatic Complications of Obesity and Alcohol”, Inserm, U1065, 06204 Nice, France; 10 Public Health Department, University of Naples Federico II, 80131 Naples, Italy; vincenzo.pilone@unina.it

**Keywords:** obesity, tirzepatide, low-energy ketogenic therapy, body composition, muscle strength, resting metabolic rate

## Abstract

**Background:** Tirzepatide (TZP), a unimolecular dual agonist targeting glucose-dependent insulinotropic polypeptide and glucagon-like peptide-1 receptors, is a promising weight loss agent in obesity. The preservation of metabolically active fat-free mass (FFM), muscle strength (MS), and resting metabolic rate (RMR) is essential for optimizing fat mass (FM) reduction. Although TZP is typically combined with a low-calorie diet (LCD), its impact on FFM is uncertain, and studies on MS and RMR are lacking. Evidence suggests that Low-Energy Ketogenic Therapy (LEKT) may reduce FM while preserving FFM, MS, and RMR. Therefore, this study aimed to compare the effects of an LEKT and an LCD, both combined with TZP, on body weight (BW), FM, FFM, MS, and RMR in patients with obesity. **Methods:** We prospectively compared the effects of TZP combined with either an LCD or LEKT in 60 patients with obesity (n = 30 per group) over 12 weeks. BW, FM, FFM, MS, and RMR were measured at baseline and after 12 weeks. Clinical parameters, an assessment of dietary compliance, and side effects were also evaluated. **Results:** At 12-week follow-up, both groups showed a significant BW reduction from baseline (TZP+LEKT, *p* = 0.0289; TZP+LCD, *p* = 0.0278), with no significant intergroup difference (*p* = 0.665). Similarly, FM decreased significantly in both cohorts (TZP+LEKT, *p* < 0.001; TZP+LCD, *p* = 0.0185), with the TZP+LEKT group achieving a greater FM loss (*p* = 0.042). However, the TZP+LCD group exhibited significant declines from baseline in FFM (*p* = 0.0284), MS (*p* = 0.0341), and RMR (*p* < 0.001), whereas we did not observe any significant changes in FFM (*p* = 0.487), MS (*p* = 0.691), and RMR (*p* = 0.263) in the TZP+LEKT group. Intergroup direct comparisons confirmed that the TZP+LCD group experienced significantly greater reductions in FFM (*p* = 0.0388), MS (*p* = 0.046), and RMR (*p* = 0.019). **Conclusions:** Based on the findings of these preliminary data, we are able to support the hypothesis that TZP+LEKT seems to be superior to TZP+LCD in promoting FM reduction while preserving FFM, MS, and RMR in patients with obesity.

## 1. Introduction

Obesity is a chronic, progressive, and recurrent disease marked by an excessive fat accumulation that adversely affects health, currently impacting approximately 650 million individuals globally [1,2]. This condition substantially heightens the risk of various metabolic complications, including type 2 diabetes (T2D), hypertension, dyslipidemia, cardiovascular diseases, chronic kidney disease, and metabolic dysfunction-associated steatotic liver disease. Additionally, obesity predisposes individuals to certain malignancies and mechanical disorders such as obstructive sleep apnea and osteoarthritis [3,4,5]. 

Given its complex and multifactorial nature, obesity management requires a comprehensive therapeutic strategy that integrates lifestyle modifications, dietary approaches, pharmacotherapy, and bariatric surgery (BS) [6]. Among these, BS remains the most effective intervention, producing substantial and sustained weight loss compared to conventional methods [7,8]. However, its scalability at the population level is limited, and some patients may be ineligible for surgery due to medical conditions, while others may be reluctant due to concerns regarding potential postoperative complications [9]. 

In recent years, a new class of anti-obesity drugs, including tirzepatide (TZP), has transformed the landscape of obesity treatment and is increasingly utilized in clinical practice [10]. TZP is a dual agonist of glucose-dependent insulinotropic polypeptide (GIP) and glucagon-like peptide-1 (GLP-1) receptors, exerting synergistic effects on appetite regulation, food intake, and metabolic function [10,11,12,13]. Approved for use in multiple countries, including Italy, TZP is administered as a once-weekly subcutaneous injection for obesity management. Standard clinical guidelines recommend that patients undergoing TZP therapy adhere to a low-calorie diet (LCD) with a daily caloric deficit of 500 kcal [14,15,16,17]. 

From a clinical perspective, the primary objective of weight reduction, including pharmacologically induced weight loss, is the reduction of body weight (BW) and fat mass (FM). However, similar to BS, incretin receptor agonists such as TZP induce a 15–25% weight loss, with over 25% of the lost weight derived from fat-free mass (FFM), which may negatively impact the metabolism and may lead to physical frailty [18]. 

Preserving an adequate FFM is critical during weight loss, as muscle tissue plays a fundamental role in whole-body protein metabolism [19]. A substantial decline in FFM may negatively affect the resting metabolic rate (RMR) [20], slow weight loss progression, increase susceptibility to weight regain [21], and heighten the risk of muscle strength (MS) loss and sarcopenia [22,23]. Therefore, quantifying FFM loss as a proportion of total weight reduction serves as a crucial indicator of the safety and effectiveness of different weight loss interventions. 

A recent systematic review of randomized controlled trials investigating the effects of TZP on body composition in individuals with overweight and obesity has demonstrated that TZP significantly reduces total FM, visceral adipose tissue, and waist circumference over short- and intermediate-term follow-up periods [24]. However, the impact of TZP on FFM loss remains inconclusive, as findings from the existing studies are inconsistent. In the SURMOUNT-1 trial, where 2539 participants were randomized to receive 5, 10, or 15 mg of tirzepatide for 72 weeks, in conjunction with a 500 kcal/day deficit diet, weight loss was accompanied by a 33.9% reduction in total FM and a 10.9% decrease in total FFM [14]. Consequently, preventive measures should be implemented early during TZP therapy to mitigate FFM loss and preserve both MS and RMR. 

Low-Energy Ketogenic Therapy (LEKT) is a structured, multi-phase dietary intervention characterized by carbohydrate restriction (<30 g/day), which induces ketosis and facilitates substantial weight loss [25,26,27]. Evidence suggests that LEKT enhances glycemic control, improves lipid profiles, and reduces the blood pressure, making it a promising dietary strategy for T2D management and cardiovascular risk reduction. Additionally, growing evidence suggests that LEKT strategies provide metabolic benefits beyond FM reduction. Unlike conventional LCD, LEKT has been shown to better preserve the FFM and RMR, both of which are often compromised during caloric restriction. Since maintaining FFM and preventing metabolic adaptation are critical challenges in obesity treatment, LEKT represents a promising dietary strategy for improving weight management outcomes [28,29,30,31]. However, one of the primary concerns regarding LEKT is the potential for increased protein catabolism due to reduced carbohydrate availability. Nonetheless, emerging evidence suggests that ketosis may have a muscle-preserving effect. Indeed, ketone bodies serve as an alternative energy substrate, reducing the reliance on gluconeogenesis from amino acids and thereby potentially mitigating muscle proteolysis [32,33]. Additionally, ketogenic diets have been shown to enhance mitochondrial efficiency, promote lipid oxidation, and modulate anabolic and catabolic pathways, which may contribute to preserving FFM during energy restriction [34,35]. 

To date, no comparative studies have evaluated the differential effects of LEKT versus standard LCD on weight loss, FM, FFM, MS, and RMR in patients undergoing TZP therapy. Therefore, this study aims to prospectively examine the distinct impacts of these dietary strategies on body composition and metabolic outcomes in two patient cohorts receiving either LEKT or LCD in combination with TZP treatment.

## 2. Materials and Methods

### 2.1. Study Design and Patient Selection

Between October and December 2024, we conducted a pilot, prospective trial involving a cohort of 60 consecutive patients with obesity undergoing TZP treatment. Participants were eligible if they were 18 years or older, had a body mass index (BMI) of ≥30 kg/m^2^, or had a BMI of ≥27 kg/m^2^ with at least one weight-related comorbidity (e.g.,T2D, hypertension, dyslipidemia, obstructive sleep apnea, or cardiovascular disease) and had previously experienced at least one unsuccessful dietary attempt to lose weight [14,15,16,17].

In alignment with previous studies on TZP therapy, major exclusion criteria included type 1 diabetes, a body weight fluctuation of more than 5 kg within the 90 days prior to screening, prior or planned bariatric surgery, and the use of weight loss medications within 90 days before study enrollment [14,15,16,17]. Additional exclusion criteria encompassed confirmed pregnancy, diagnosed eating disorders (e.g., bulimia, binge eating disorder, night eating syndrome), serum creatinine levels exceeding 1.8 mg/dL, liver enzyme levels (glutamic-oxaloacetic transaminase (GOT) or glutamic-pyruvic transaminase (GPT)) exceeding three times the upper normal limit, an inability to adhere to either the LEKT or LCD due to religious restrictions, and the presence of chewing or swallowing impairments [26,29,31]. All procedures conducted in this study adhered to the ethical guidelines set forth by the institutional and/or national research committee, in accordance with the 1964 Declaration of Helsinki and its subsequent amendments. This study did not require formal approval from an ethics committee, as it was classified under the “negligible risk research” category—defined as research posing no foreseeable risk of harm or discomfort.

**Figure 1:**
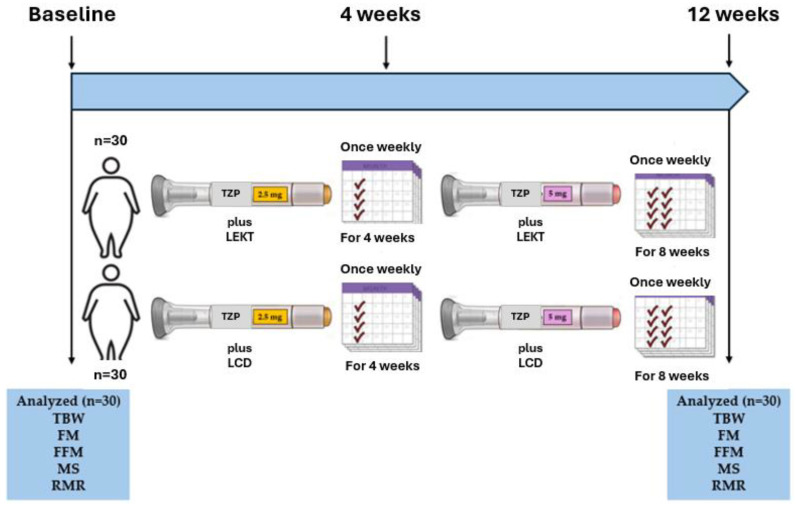
Flow chart comparing a 12-week TZP+LEKT vs. TZP+LCD. LEKT = Low-Energy Ketogenic Therapy; TZP = Tirzepatide; LEC = low-calorie diet; TBW = total body weight; FM = fat mass; FFM = fat-free mass; MS = muscle strength; RMR = resting metabolic rate.

Furthermore, the TZP utilized in this study is not an experimental drug but is already approved by both the U.S. Food and Drug Administration and the Italian Medicines Agency (AIFA) for obesity treatment. As such, it has received full authorization for prescription in patients with obesity. Additionally, numerous studies have confirmed the safety and efficacy of LEKT in reducing BW and BMI, as well as in improving clinical outcomes in individuals with obesity [36,37,38,39]. Before initiating TZP therapy, all participants underwent individual counseling regarding the dietary regimen they would follow for 12 weeks. They were subsequently allocated to either the LEKT or LCD group based on their feasibility and commitment to adhering to the respective dietary protocols. Given that individual personality, cognitive maturity, knowledge, and comprehension are key determinants influencing adherence to the prescribed diets, we opted for a non-randomized allocation approach. Patients were assigned to the group deemed most appropriate for their specific conditions and adherence capacity (Figure 1). Although randomization is the gold standard for minimizing bias, we opted for a non-randomized allocation based on patients’ ability and willingness to adhere to the assigned dietary protocol. Adherence is a key determinant of dietary intervention success, as highlighted in previous research [40,41]. Consequently, two study groups were established: the TZP+LEKT group (n = 30) and TZP+LCD group (n = 30). The AIFA has approved TZP for obesity treatment in six dosage levels (2.5 mg, 5 mg, 7.5 mg, 10 mg, 12.5 mg, and 15 mg). However, at the time of the study, only two doses (2.5 mg and 5 mg) were available for use in Italy. Written informed consent was obtained from each participant following a detailed explanation of the study’s purpose and methodology.

### 2.2. TZP Protocol and LEKT and LCD Characteristics

As illustrated in Figure 1, all participants in the study adhered to the following treatment protocol: TZP was initially administered at a dose of 2.5 mg subcutaneously once per week for four weeks, in conjunction with either LEKT or LCD. This was followed by an increase in TZP dosage to 5 mg per week for an additional eight weeks, maintaining the respective dietary regimen. To ensure a uniform dietary intake among all participants, two standardized meal plans were developed, one for LEKT and one for LCD. The LEKT meal plan was structured using a free online ketogenic diet application ([https://www.eatthismuch.com]), while the LCD meal plan was formulated using Nutrigeo 8 software (Progeo, Ascoli Piceno, Italy). Both tools facilitated the assignment of precise food quantities tailored to individual participants. In accordance with the SURMOUNT-1 study, the macronutrient composition of the LCD consisted of 50% carbohydrates, 20% protein, and 30% fat, totaling approximately 1200 kcal/day [14]. In contrast, the LEKT regimen, as previously documented [26,31], was characterized by less than 30 g of carbohydrates per day, 43% protein (1.3 g/kg of ideal body weight), and 44% fat, with an equivalent caloric intake of ~1200 kcal/day. Throughout the 12-week follow-up period, participants were not encouraged to alter their physical activity levels. Evaluations were conducted at baseline (one day before the initiation of TZP therapy) and at the end of the 12-week intervention period, enabling an assessment of treatment efficacy and metabolic outcomes.

### 2.3. Assessment of BW, FM, FFM, MS, RMR, and Laboratory Parameters

BW (kg) and height (cm) were measured under standardized conditions. Height was determined using a Seca 206 mechanical measuring tape (Intermed, Milano, Italy), while BW was recorded using the Seca 869 flat digital scale (maximum capacity: 250 kg; Intermed, Milano, Italy).

Body composition was assessed through bioelectrical impedance analysis (BIA) utilizing the Jawon IOI 353 Segmental Body Composition Monitor (Cosmed, Rome, Italy) [26,31,38]. This device represents a state-of-the-art advancement in body composition analysis, employing multi-frequency technology and complying with Directive 90/384/EEC (for non-automatic weighing instruments in the medical sector) and Directive 93/42/EEC (for medical devices).

To ensure accurate BIA measurements, participants were required to adhere to specific pre-assessment conditions, as previously reported [26,31,42]. These conditions included the following: abstaining from food intake for at least 4 h before testing, consuming a minimum of 2 liters of water the day before the examination, avoiding physical activity for at least 8 h prior to the assessment, refraining from coffee or alcoholic beverage consumption for at least 12 h, not using diuretics within 24 h before the test, and emptying the bladder immediately before undergoing BIA.

RMR was determined using indirect calorimetry with the Fitmate PRO monitor (Cosmed, Italy) [31,43,44]. Measurements were conducted between 8:00 and 10:00 AM in a temperature-controlled room, minimizing diurnal fluctuations between participants [45,46,47,48]. RMR was measured following standardized guidelines [48]. To ensure accurate measurements, participants were instructed to fast for at least 4 h, avoid caffeine and alcohol for at least 12 h, and refrain from physical activity for at least 24 h before the assessment. Measurements were conducted in a supine position within a thermoneutral environment and lasted 15 min. To enhance reliability, the last 5 min of data were used for analysis, excluding the initial stabilization phase. Energy expenditure was calculated based on oxygen consumption (VO_2_) and the respiratory quotient, according to established methodologies [48].

MS was evaluated using a hand dynamometer, measuring absolute handgrip strength in the dominant hand. Each participant was seated with the arm flexed at 90° and instructed to exert their maximum grip strength for 3 s, with 15 s rest intervals between trials. The highest recorded force (kg) was designated as absolute MS [22,49].

Laboratory analyses included the assessment of liver enzymes (GOT, GPT, and gamma-glutamyl transferase [GGT]), glucose, insulin, creatinine, urea, uric acid, blood urea nitrogen, ketonemia, iron, hemoglobin, total cholesterol, high-density lipoprotein (HDL), low-density lipoprotein (LDL), and triglycerides. All biochemical tests were conducted in a certified laboratory, adhering to both internal and external quality control procedures and following the manufacturer’s standardized protocols.

### 2.4. Evaluation of Treatment Adherence and Side Effects

Adherence to both the dietary protocols and TZP therapy was systematically monitored throughout the study. Regarding TZP therapy, adherence was strictly monitored, as patients were required to attend hospital visits for drug administration. This ensured full compliance and allowed for close supervision by healthcare professionals, minimizing variability in treatment adherence. Regarding dietary adherence, in the LEKT group, compliance was assessed by measuring blood ketone levels, ensuring that participants remained in nutritional ketosis. Additionally, WL progression was monitored in both groups as a secondary adherence measure. These adherence-monitoring strategies ensured the reliability of both the dietary and pharmacological interventions throughout the study. 

To monitor potential dietary side effects, a combination of qualitative methods was employed. During monthly counseling sessions, participants were required to complete a standardized questionnaire designed to evaluate appetite fluctuations and document the occurrence of adverse symptoms, including nausea, vomiting, diarrhea, and constipation [14,15,16,17]. A certified nutritionist conducted a thorough review of all the submitted questionnaires, ensuring accuracy, completeness, and consistency in the reported data.

### 2.5. Statistical Analysis

The effects of TZP+LEKT compared to TZP+LCD on BW, FM, FFM, MS, and RMR were assessed using paired-samples *t*-tests for within-group comparisons and Mann–Whitney tests for between-group analyses. All statistical analyses were performed using GraphPad Prism for Windows (version 9.1.2p, GraphPad Software, La Jolla, CA, USA). Statistical tests were selected based on data distribution and sample size. The Shapiro–Wilk test confirmed normality (*p* > 0.05), justifying the use of paired *t*-tests for within-group comparisons. However, given the relatively small sample size (n = 30 per group) and a slight skewness in some variables, the Mann–Whitney U test was preferred for between-group comparisons to enhance the robustness and minimize bias. This combined approach ensured a reliable assessment of the differential effects of TZP+LEKT and TZP+LCD on body composition and metabolic parameters.

Data are presented as the mean ± standard deviation (SD), with a *p*-value < 0.05 considered statistically significant. Additionally, any *p*-value lower than 0.001 was conventionally reported as *p* < 0.001. Changes in BW, BMI, FM, FFM, MS, and RMR were expressed as percent variations and plotted over time. 

Furthermore, a two-way ANOVA was performed to evaluate the independent and interactive effects of the dietary intervention (LEKT vs. LCD) and time (baseline vs. 12-week follow-up) on FFM. Additionally, to assess differences in FFM retention, we calculated the percentage change in FFM relative to baseline using the following formula:

$$\%\Delta FFM=\frac{\left(FFM\;final-FFM\;baseline\right)}{FFM\;baseline}\;\times \;100$$

Data normality was confirmed using the Shapiro–Wilk test (*p* > 0.05 for both groups). Differences between groups were analyzed using an independent *t*-test.

## 3. Results 

### 3.1. Characteristics of the Study Groups at Baseline

The study included 60 patients (33 females and 27 males). Before TZP treatment, the TZP+LEKT and TZP+LCD groups were comparable in terms of BW, BMI, FM, FFM, MS, and RMR (Table 1).

### 3.2. Impact of TZP+LEKT vs. TZP+LCD on BW, FM, FFM, MS, and RMR

**Figure 2:**
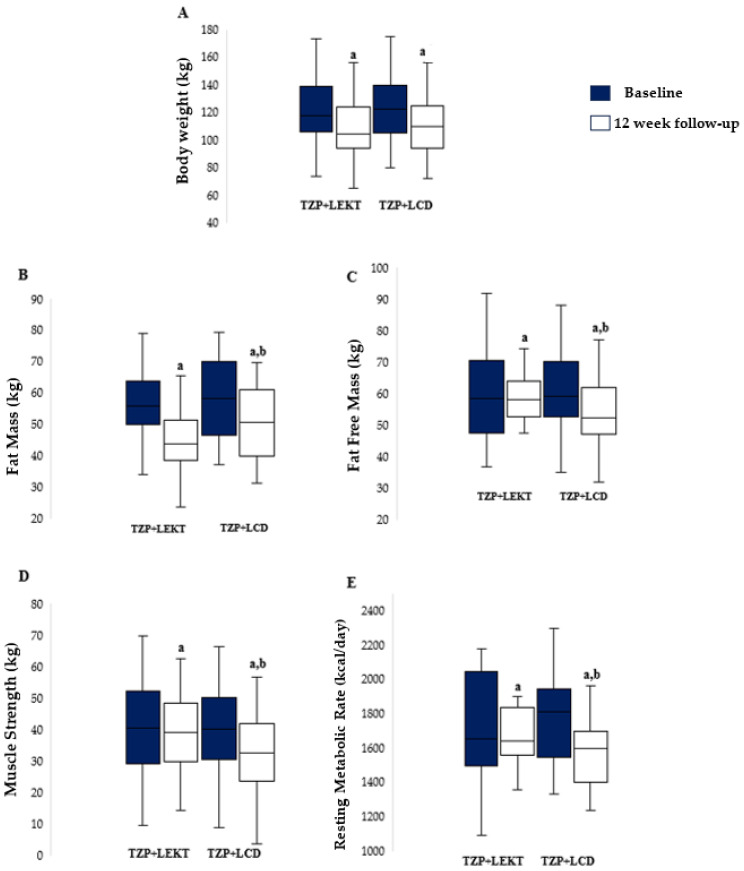
Box plots show baseline and 12-week follow-up changes in body weight (**A**), fat mass (**B**), fat-free mass (**C**), muscle strength (**D**), and resting metabolic rate (**E**) in both groups studied. Horizontal bars represent the median values. Lower and upper boundaries of the box represent the first and the third quartile, respectively. Lower and upper error bars represent the minimum and the maximum value, respectively. TZP= tirzepatide; LEKT= Low-Energy Ketogenic Therapy; LEC= low-calorie diet. ^a^ 12 weeks follow-up vs. baseline; ^b^ 12 weeks follow-up TZP+LEKT vs. LCD.

No patients dropped out during the study. At the 12-week follow-up, both groups showed a significant reduction in BW compared to baseline (TZP+LEKT, *p* = 0.0289; TZP+LCD, *p* = 0.0278), with no significant difference between them (*p* = 0.665). The percentage decrease in BW was −10.2% ± 2.5 in the TZP+LEKT group and −9.8% ± 2.9 in the TZP+LCD group (Figure 2A). Both groups also experienced a significant reduction in FM (TZP+LEKT, *p* < 0.001; TZP+LCD, *p* = 0.0185), though the decrease was significantly greater in the TZP+LEKT group (−13.4% ± 2.8 vs. −10.2% ± 3.1, *p* = 0.042) (Figure 2B). Similarly, a significant reduction in FFM was observed in both groups, but the decline was significantly greater in the TZP+LCD group (−4.29% ± 1.31) compared to the TZP+LEKT group (−0.50% ± 0.82, *p* = 0.0388) (Figure 2C). MS also declined significantly in both groups (TZP+LCD, *p* = 0.0341), with a greater reduction in the TZP+LCD group (−4.1% ± 1.2) compared to the TZP+LEKT group (−0.3% ± 0.9, *p* = 0.046) (Figure 2D). Regarding RMR, a significant reduction was observed only in the TZP+LCD group (−5.3% ± 1.8, *p* < 0.001), whereas the decrease in the TZP+LEKT group (−1.2% ± 0.9, *p* = 0.263) was not statistically significant. The intergroup comparison confirmed that the reduction in RMR was significantly smaller in the TZP+LEKT group (*p* = 0.019) (Figure 2E). A two-way ANOVA revealed that neither the main effect of the dietary intervention (*p* = 0.240) nor time (*p* = 0.967) reached statistical significance. However, the interaction term (Group × Time) approached significance (*p* = 0.088), suggesting a potential trend toward a differential effect on FFM over time.

### 3.3. Impact of TZP+LEKT vs. TZP+LCD on Patient’s Clinical Parameters

As reported in Table 2, we found a significant amelioration of the general clinical status in both groups studied. However, we observed a greater and significant improvement in HOMA Index, hemoglobin A1C, total cholesterol, and triglycerides in the TZP+LEKT group in comparison with TZP+LCD group. Furthermore, as expected, at the time of follow-up, ketonemia levels were significantly higher in the TZP+LEKT group than in the TZP+LCD group.

### 3.4. Impact of TZP+LEKT vs. TZP+LCD on Patient’s Appetite and Side Effects

Interestingly, a greater proportion of participants in the TZP+LEKT group reported a decrease in appetite compared to those in the TZP+LCD group (60% vs. 26.7%). This effect may be attributed to the ketogenic dietary regimen, which is often associated with reduced hunger perception. Regarding adverse events, nausea was reported by 50% of participants in the TZP+LEKT group and 56.7% in the TZP+LCD group, while constipation was experienced by 53.3% and 50%, respectively. Vomiting occurred in 20% of participants in the TZP+LEKT group and 23.3% in the TZP+LCD group. Additionally, diarrhea was the least frequent adverse event, affecting 6.7% of participants in the TZP+LEKT group and 10% in the TZP+LCD group (Table 3).

Overall, both dietary interventions were well tolerated, with no severe adverse reactions reported. The frequency of mild gastrointestinal symptoms was similar between groups, suggesting that the observed effects were primarily related to the pharmacological treatment rather than the dietary regimen.

## 4. Discussion

Based on the findings of these preliminary data, we are able to support the hypothesis that TZP+LEKT seems to be superior to TZP+LCD in promoting FM reduction while preserving FFM, MS, and RMR in patients with obesity. 

The preservation of FFM, MS and RMR in the TZP+LEKT group may be explained by ketosis-induced metabolic adaptations. Ketone bodies, particularly β-hydroxybutyrate, act as alternative energy substrates, reducing muscle proteolysis and promoting protein sparing [32,33]. Ketosis also enhances mitochondrial efficiency, sustaining ATP production and preventing metabolic slowdown [34,35]. 

Additionally, ketogenic diets have been shown to modulate the secretion of various myokines involved in metabolic regulation and muscle preservation. Contrary to earlier hypotheses, recent evidence indicates that FGF21 levels actually decrease in response to ketogenic diets and energy restriction and may serve more as a marker of metabolic stress than as a direct effector of muscle maintenance [50,51].

Conversely, other myokines such as irisin and interleukin-6 appear to be positively modulated by ketogenic diets and may contribute to the preservation of FFM and metabolic flexibility during WL in individuals with obesity [52]

The SURMOUNT clinical trial program (SURMOUNT-1, 2, 3, and 4) represents the most extensive evaluation of TZP in obesity management [14,15,16,17]. We selected SURMOUNT-1 [14] as our primary reference because it included only non-diabetic individuals, making it more comparable to our study population. Although SURMOUNT-1 was a 72-week trial, we extrapolated 12-week weight loss, FM, and FFM reductions to align with our study timeframe. In contrast, SURMOUNT-2 assessed TZP in individuals with obesity and T2D, introducing metabolic differences that could confound direct comparisons [15]. Meanwhile, SURMOUNT-3 and SURMOUNT-4 involved participants with prior weight loss interventions (SURMOUNT-3) or intensive lifestyle modifications (SURMOUNT-4), differing from our study design, where no structured weight loss preceded tirzepatide initiation [16,17]. In SURMOUNT-1, patients receiving TZP 5 mg experienced an estimated 7.5% reduction in body weight at 12 weeks [14]. In contrast, our study found that patients following TZP+LEKT achieved a 10.2% reduction in body weight, while those on TZP+LCD experienced a 9.8% reduction. These findings indicate that nutritional interventions could enhance the early efficacy of TZP treatment. Additionally, the greater weight reduction observed with TZP+LEKT compared to TZP+LCD suggests that ketosis-induced metabolic adaptations, such as enhanced fat oxidation and appetite suppression, may contribute to a more rapid weight loss response [53,54].

When analyzing FM loss, SURMOUNT-1 estimated a 12-week FM reduction of 8.5% with TZP 5 mg [14]. In comparison, TZP+LEKT achieved a 13.4% reduction, while TZP+LCD resulted in a 10.2% decrease. These results demonstrate that combining TZP with either dietary approach effectively promotes FM loss. However, the more pronounced FM reduction observed with TZP+LEKT suggests that ketogenic therapy may offer an additional advantage in selectively targeting fat loss while preserving metabolically active tissues [55]. 

Regarding FFM, in SURMOUNT-1, patients receiving TZP 5 mg experienced an estimated 1.9% reduction in body weight at 12 weeks [14]. In our study, the TZP+LEKT group had a minimal FFM loss of −0.5%, whereas the TZP+LCD group exhibited a greater FFM loss of −4.29%, confirming that ketogenic therapy may play a crucial role in maintaining muscle integrity and preventing metabolic adaptation, two common concerns during weight loss interventions [55,56,57]. 

Although the ANOVA did not confirm a statistically significant effect, the percentage differences suggest that the LEKT approach may have contributed to better FFM preservation.

While SURMOUNT-1 primarily assessed BW, FM, and FFM, our study represents the first investigation to comprehensively evaluate the impact of TZP-based therapy on both MS and RMR. This novel analysis indicates that the LEKT approach enhances FM loss while preserving muscle function and metabolic rate, two key determinants of long-term weight management and metabolic health [58,59]. Our findings indicate that patients in the TZP+LCD group experienced a significant decline in MS (−4.1%) and RMR (−5.3%), highlighting the adverse effects of FFM loss on muscle function and metabolic activity. Conversely, TZP+LEKT preserved MS (−0.3%) and RMR (−1.2%), further supporting the protective role of ketogenic nutritional strategies in mitigating both muscular and metabolic decline [55,56,57]. 

Several biochemical and molecular pathways are hypothesized to contribute to the preservation of FFM during weight loss, playing a critical role in mitigating declines in MS and RMR. Skeletal muscle sustains MS through activation of the phosphoinositide 3-kinase (PI3K)/Akt/mammalian target of rapamycin pathway, which promotes protein synthesis while inhibiting proteolysis via Forkhead box O transcription factors [60,61]. Additionally, the activation of AMP-activated protein kinase and peroxisome proliferator-activated receptor gamma coactivator 1-alpha enhances mitochondrial biogenesis, thereby optimizing energy efficiency [62,63,64]. The maintenance of FFM further supports RMR by preserving mitochondrial density and oxidative metabolism, ensuring sustained ATP production and thermogenesis through the uncoupling of proteins [65,66]. 

Although the optimal duration of a LEKT remains a subject of debate, we also report here on the safety of a 12-week LEKT, which is consistent with several clinical trials demonstrating its effectiveness and safety for weight loss and metabolic health. Indeed, a randomized controlled trial in females with obesity and overweight, who had glucose and lipid metabolism disturbances, reported significant reductions in body weight and improvements in biochemical markers without adverse effects over 12 weeks [67]. Furthermore, Dashti et al. conducted a study to assess the long-term effects of a 24-week ketogenic diet in patients with obesity. The findings demonstrated significant reductions in body weight and improvements in overall clinical status. Importantly, the study concluded that administering a ketogenic diet for a relatively extended period did not produce any significant side effects, corroborating its safety for long-term use [68]. Similarly, a study on individuals with obesity and knee osteoarthritis found that a 20-week LEKT was well tolerated and led to substantial weight loss and clinical benefits, further reinforcing its short-term safety [69,70]. These findings suggest that while the long-term safety of LEKT requires further investigation, a 12-week intervention remains a viable and effective dietary strategy for obesity management. 

Additionally, in SURMOUNT-1, the most commonly reported adverse events included gastrointestinal symptoms such as nausea (17.8%), diarrhea (13.5%), and vomiting (6.2%) in patients receiving tirzepatide 5 mg [14]. Our study observed a lower incidence of gastrointestinal side effects in the TZP+LEKT group, possibly due to ketosis-induced metabolic adaptations, which could improve gastrointestinal tolerance [53]. However, further studies are needed to confirm this hypothesis.

This study has some limitations that should be considered. First, its non-randomized design. While randomization is the gold standard for minimizing bias, this study employed a non-randomized design to prioritize patient adherence to the assigned dietary protocol. Since adherence is a key determinant of dietary intervention success, this approach was chosen to enhance compliance and ensure feasibility. Importantly, baseline characteristics were well balanced between groups (Table 1), suggesting that major confounding factors were unlikely to have significantly influenced the results. Nevertheless, future studies should incorporate randomized designs to further strengthen the validity of their findings. 

In our study, participants were assigned to LEKT or LCD based on their ability and willingness to follow the dietary protocols, potentially influencing baseline characteristics such as motivation, metabolic flexibility, and prior dietary experience. Although randomized controlled trials minimize bias by even distributing confounders, real-world studies suggest that adherence is a critical determinant of dietary intervention success. Our approach prioritizes real-world feasibility, which may enhance long-term compliance [40,41]. This study was designed as a pilot investigation to provide preliminary evidence on the metabolic and body composition effects of LEKT versus LCD in patients undergoing tirzepatide therapy. Given its exploratory nature, no a priori power calculation was performed, as the aim was to assess feasibility rather than to establish definitive statistical significance. Similarly, a post hoc power analysis was not conducted, as it would not provide meaningful insights in a pilot study. We acknowledge that the small sample size and short intervention duration may limit the statistical significance, and these findings should be confirmed in larger, adequately powered trials. In the present study, participants were instructed to maintain their usual activity levels. While we acknowledge that physical activity is a key factor in weight management, body composition, and metabolic health, we intentionally did not provide any specific recommendations to avoid introducing an additional confounding variable. Our primary objective was to specifically evaluate the potential metabolic and body composition effects of two different dietary approaches (LEKT vs. LCD) in combination with TZP, independent of variations in exercise habits. Including physical activity as an additional variable could have influenced the interpretation of our results and masked the specific effects of the dietary interventions. Future studies should incorporate objective measures of physical activity, such as accelerometers or structured exercise protocols, to better delineate the independent and combined effects of dietary and physical activity interventions on metabolic health and to explore how diet and exercise interact in obesity treatment and metabolic regulation.

## 5. Conclusions

Based on the findings of these preliminary data, we are able to support the hypothesis that TZP+LEKT seems to be superior to TZP+LCD in promoting FM reduction while preserving FFM, MS, and RMR in patients with obesity. These findings highlight the potential of structured LEKT strategies to enhance TZP’s efficacy. However, further long-term studies are necessary to confirm these effects.

## Figures and Tables

**Table 1:** Characteristics of study groups at baseline. Data are reported as mean ± SD.

	LEKT (N = 30)	LCD (N = 30)	*p* (LEKT vs. LCD)
Sex (male/female, n)	14/16	13/17	-
Body Weight (kg)	121.9 ± 23.6	124.3 ± 23.8	0.70
Body Mass Index (kg/m^2^)	44.9 ± 6.28	45.5 ± 7.35	0.73
Fat Mass (kg)	56.4 ± 11.6	58.4 ± 13	0.53
Fat Free Mass (kg)	59.9 ± 14.3	60.6 ± 12.8	0.84
Muscle Strength (kg)	41 ± 15	40.5 ± 14.1	0.79
Resting Metabolic Rate (kcal/day)	1716 ± 317	1769 ± 235	0.46

LEKT = Low-Energy Ketogenic Therapy. LEC = low-calorie diet. SD = standard deviation.

**Table 2:** Patients’ clinical parameters at baseline and after 12 week. Data are reported as mean ± SD.

Clinical Parameters (Cut-Off)	Group	Baseline	12-Week Follow-Up	*p*
Glucose (70–100 mg/dL)	TZP+LEKT	87 ± 21	80 ± 18	0.17
	TZP+LCD	92 ± 17	87 ± 13	0.21
Insulin (<25 mU/L)	TZP+LEKT	8.5 ± 6.3	7.1 ± 5.2	0.35
	TZP+LCD	7.7 ± 4.7	7.2 ± 3.1	0.63
HOMA Index (<2.5)	TZP+LEKT	1.83 ± 0.8	1.40 ± 1.1	0.09 *
	TZP+LCD	1.75 ± 1.4	1.55 ± 0.96	0.52
Hemoglobin A1C (<6.1%)	TZP+LEKT	4.7 ± 1.7	3.7 ± 1.1	0.01 *
	TZP+LCD	4.3 ± 0.77	4.0 ± 0.80	0.14
Creatine (0.55–1.02 mg/dL)	TZP+LEKT	0.76 ± 0.19	0.81 ± 0.26	0.40
	TZP+LCD	0.79 ± 0.17	0.83 ± 0.15	0.34
Iron (50–170 μg/dL)	TZP+LEKT	59.3 ± 34	60 ± 24	0.93
	TZP+LCD	68 ± 19	63.5 ± 21	0.39
Uric acid (3.0–7.0 mg/dL)	TZP+LEKT	5.3 ± 1.2	5.7 ± 2.2	0.39
	TZP+LCD	5.4 ± 1.4	5.1 ± 1.3	0.39
Total cholesterol (<200 mg/dL)	TZP+LEKT	194 ± 42	162 ± 11	<0.001 *
	TZP+LCD	176 ± 48	159 ± 23	0.09
HDL (>50 mg/dL)	TZP+LEKT	47 ± 12	55.5 ± 18	0.03
	TZP+LCD	49.9 ± 22	45.4 ± 10	0.31
Triglycerides (<150 mg/dL)	TZP+LEKT	151 ± 75	118 ± 28.5	0.03 *
	TZP+LCD	116 ± 54	109 ± 18.2	0.50
GOT (<34 U/L)	TZP+LEKT	23 ± 13.8	24 ± 8.2	0.73
	TZP+LCD	26 ± 22.9	29 ± 9.5	0.51
GPT (<55 U/L)	TZP+LEKT	29 ± 21.1	19 ± 3.7	0.01
	TZP+LCD	35 ± 32.4	29 ± 8.7	0.33
GGT (<38 U/L)	TZP+LEKT	32 ± 26.4	28 ± 6.9	0.42
	TZP+LCD	31 ± 21.1	29 ± 9.2	0.64
Na^2+^ (13–146 mmol/L)	TZP+LEKT	134 ± 33.7	132 ± 32.2	0.81
	TZP+LCD	136 ± 32.3	135 ± 35.2	0.91
K^+^ (3.4–5.1 mmol/L)	TZP+LEKT	4.1 ± 0.48	3.9 ± 0.4	0.08
	TZP+LCD	4.2 ± 0.44	4 ± 0.7	0.19
Cl^2−^ (101–110 mmol/L)	TZP+LEKT	103 ± 6.3	101 ± 5.4	0.19
	TZP+LCD	106 ± 2.7	105 ± 3.5	0.22
Ketonemia (<0.6 mmol/L)	TZP+LEKT	0.3 ± 0.04	0.82 ± 0.49	<0.001
	TZP+LCD	0.25 ± 0.07	0.27 ± 0.05	0.21

HOMA Index = homeostasis model assessment; HDL = high-density Lipoprotein; LDL = low-density lipoprotein; GOT = glutamic-oxaloacetic transaminase; GPT = glutamate-pyruvate transaminase; GGT = gamma-glutamyl transferase; * 12 weeks follow-up vs. baseline.

**Table 3:** Self-reported rates regarding a decrease in appetite, nausea, vomiting, constipation, and diarrhea over the 12-week period of the TZP treatment in both groups studied.

Symptoms	LEKT (N = 30)	LCD (N = 30)
Decreased appetite, (%)	18	8
Nausea, (%)	15	17
Vomiting, (%)	6	7
Constipation, (%)	16	15
Diarrhea, (%)	4	3

LEKT = Low-Energy Ketogenic Therapy. LEC = low-calorie diet.

## Data Availability

The data included in this manuscript were derived from the University database. We are not authorized to share the data with third-party organizations. However, the corresponding author is available to provide any explanation to the Editor if requested.
